# A Novel Method for Identification and Quantification of Sulfated Flavonoids in Plants by Neutral Loss Scan Mass Spectrometry

**DOI:** 10.3389/fpls.2019.00885

**Published:** 2019-07-05

**Authors:** Niklas Kleinenkuhnen, Felix Büchel, Silke C. Gerlich, Stanislav Kopriva, Sabine Metzger

**Affiliations:** ^1^MS-Platform, Cluster of Excellence on Plant Sciences, Botanical Institute (CEPLAS), University of Cologne, Cologne, Germany; ^2^Botanical Institute and Cluster of Excellence on Plant Sciences (CEPLAS), University of Cologne, Cologne, Germany

**Keywords:** mass spectrometry, sulfur metabolism, *Flaveria*, method development, sulfated flavonoids, neutral loss scan

## Abstract

Sulfur is present in plants in a large range of essential primary metabolites, as well as in numerous natural products. Many of these secondary metabolites contain sulfur in the oxidized form of organic sulfate. However, except of glucosinolates, very little is known about other classes of such sulfated metabolites, mainly because of lack of specific and quantitative analytical methods. We developed an LC-MS method to analyze sulfated flavonoids, a group of sulfated secondary metabolites prominent, e.g., in plants of the genus *Flaveria*. The method uses a linear gradient of methanol/formic acid in water on a Restek Raptor C_18_ Core-Shell column for separation of the compounds. The sulfated flavonoids are detected by mass spectrometry (MS) in a negative mode, using a neutral loss of 80 Da after a collision induced dissociation. With this method we were also able to quantify the sulfated flavonoids. We could detect all (mono)sulfated flavonoids described before in *Flaveria* plus a number of new ones, such as isorhamnetin-sulfate-glycoside. In addition, we showed that sulfated flavonoids represent a substantial sulfur pool in *Flaveria*, larger than the thiols glutathione and cysteine. The individual species possess different sulfated flavonoids, but there is no correlation between the qualitative pattern and type of photosynthesis. Similar to other sulfur-containing secondary compounds, the concentration of sulfated flavonoids in leaves is reduced by sulfur starvation. The new LC-MS method will enable qualitative and quantitative detection of these secondary metabolites in plants as a pre-requisite to addressing their functions.

## Introduction

Sulfur (S) is an essential nutrient for all life forms. In plants it is present in a plethora of metabolites of primary and secondary metabolism, most prominently in the amino acids cysteine and methionine. In the majority of these metabolites S is present in a reduced form, i.e., as a thiol, however, some compounds, mainly various secondary metabolites, contain S in an oxidized form, i.e., as a sulfate (Takahashi et al., 2011). For both groups of metabolites, the source of S is sulfate that is taken up from soil and assimilated. In the sulfate assimilation pathway, the first step is activation of sulfate by adenylation to adenosine 5′-phosphosulfate (APS) catalyzed by ATP sulfurylase. APS is the branching point between the reduced and oxidized S metabolism. APS can be reduced to sulfite and further to sulfide and incorporated into cysteine or phosphorylated to 3′ -phosphoadenosine 5-phosphosulfate (PAPS), a donor of activated sulfate for synthesis of sulfated compounds by sulfotransferases (SOT) (Takahashi et al., 2011; Hirschmann et al., 2014). The control of S partitioning in the primary (reduced) or secondary (oxidized) metabolism is achieved by the interplay of the corresponding enzymes, APS reductase and APS kinase (Mugford et al., 2011).

This and other knowledge of regulation of S metabolism has been mainly acquired in the model plant *Arabidopsis thaliana*, which produces large amounts of sulfated secondary compounds, glucosinolates (Halkier and Gershenzon, 2006; Sønderby et al., 2010). The amount of S that is incorporated into glucosinolates is similar to that incorporated into cysteine, glutathione and proteins (Mugford et al., 2011) and has thus large effect on general S balance. It is, therefore, important to confirm the regulation of S metabolism also in other plant species that do not form glucosinolates. In contrast, such investigations are hampered by the lack of detailed information of other sulfated metabolites in plants. One such class of metabolites is the sulfated flavonoids.

**FIGURE 1:**
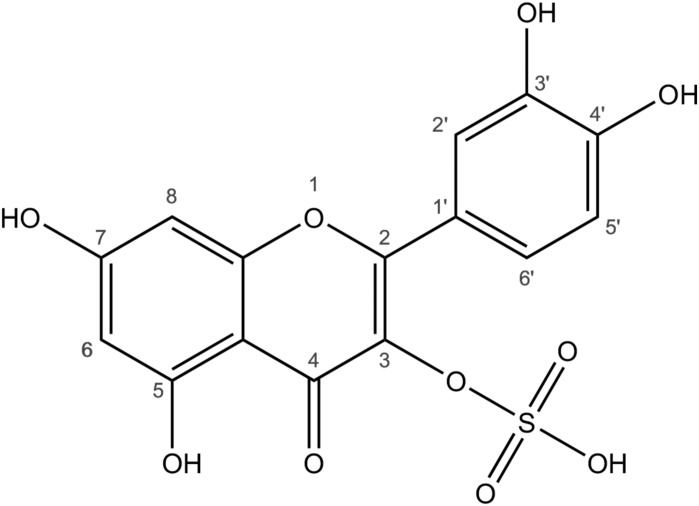
Structure of quercetin-3-sulfate. Sulfoflavonoids consist of the flavonoid specific polyphenolic C6-C3-C6 structure with one or more sulfate groups attached to the hydroxyl groups. The numbers refer to the carbon atoms and the positions of sulfations and other modifications.

Flavonoids in general are proposed to fulfill numerous functions in plants which are as diverse as their structural diversity. They are involved in plant pigmentation (Koes et al., 1994), plant microbe interactions (Hassan and Mathesius, 2012), developmental regulation (Taylor and Grotewold, 2005), and photo protection (Cao et al., 1997). In contrast the biological function(s) of sulfated flavonoids is mostly unknown. Proposed functions are co-pigmentation (Alluis and Dangles, 2001), depressing effects on cell auxin efflux (Ananvoranich et al., 1994), nuclear binding (Ibrahim, 1995), and they are discussed as part of the adaption to moist and humid habitats (Tomás-Barberán et al., 1987). Sulfated flavonoids have been reported in at least 15 monocotyledonous and 17 dicotyledonous families comprising 250 species (Barron et al., 1988; Teles et al., 2018). They usually carry one or more sulfate groups at position 3 or 7 (Figure 1). Furthermore, sulfated flavonoid glycosides have also been reported Barron et al. (1988). Flavonol specific SOTs have been described in *A. thaliana* (Gidda and Varin, 2006, Hashiguchi et al., 2014) and in several species of the genus *Flaveria* (Varin et al., 1992; Ananvoranich et al., 1994; Marsolais and Varin, 1998).

The genus *Flaveria* comprises 21 species which show a gradient of photosynthetic mechanisms ranging from C_3_ over C_3_-C_4_ intermediate to C_4_-like and C_4_ photosynthesis, making it an excellent model to study the evolution of C_4_-photosynthesis (Ku et al., 1991; Schulze et al., 2013). All species of the genus *Flaveria* produce sulfated flavonoids (Barron et al., 1988). Different *Flaveria* species, however, produce different combinations of sulfated flavonoids (Hannoufa et al., 1994). *Flaveria* has already been used to study S metabolism, particularly its adaptation to different photosynthesis types (Koprivova et al., 2001; Weckopp and Kopriva, 2014; Gerlich et al., 2018). Several important questions related to sulfated flavonoids in *Flaveria*, however, await clarification. (I) Is there a gradient in sulfated flavonoid content in *Flaveria* related to photosynthesis type similar to that seen for cysteine and glutathione (Koprivova et al., 2001; Gerlich et al., 2018)? (II) Is the content of sulfated flavonoids reduced during S starvation? (III) Do the sulfated flavonoids form a significant S pool as the glucosinolates in *Arabidopsis*?

Answering these questions is hindered by the lack of a quantitative method to measure the concentration of sulfated flavonoids in plant tissues. Previous studies were carried out using thin-layer chromatography (TLC), electrophoresis, ^13^C-NMR spectroscopy or derivatization driven UV/Vis high performance liquid chromatography (HPLC) analyses (Varin et al., 1986; Barron et al., 1988; Teles et al., 2018). Those methods require extensive sample preparation and are limited in the detection of novel compounds where no analytical standards exist. ^13^C-NMR spectroscopy needs high amounts of initial plant material for identification. Quantification could previously only be achieved by HPLC-UV/Vis but this method lacked the possibility of the identification of unknown compounds (Barron et al., 1988; Teles et al., 2018). Therefore, we explored whether recent advances in HPLC and mass spectrometry (MS) may allow new approaches to answer the questions about sulfated flavonoids and their functions.

Here we present a method for a rapid identification and absolute quantification of (mono)sulfated flavonoids from plant tissues based on a neutral loss scan MS method. With this method we analyzed the effect of S starvation on sulfated flavonoids in six *Flaveria* species with different photosynthetic mechanisms ranging from C_3_ to C_3_-C_4_, C_4_-like and C_4_ photosynthesis.

## Materials and Methods

### Chemicals and Plant Material

All solvents in the extraction and quantification were of analytical grade. The deuterated sakuranetin used as internal standard was synthesized by the group of Prof. Dr. H. G. Schmalz, Department of Chemistry, University of Cologne (Jaegle et al., 2016). The synthetic quercetin-3-sulfate and quercetin-7-sulfate used for method development and quantification were kindly provided by Frédéric Marsolais (Department of Biology, University of Western Ontario, London, Canada).

### Plant Cultivation

*Flaveria* (*F. robusta*, *F. pringlei*, *F. linearis*, *F. anomala*, *F. australasica*, and *F. bidentis*) seeds were sterilized for 4 h with chlorine gas. Stratified seeds were plated on ½ Murashige Skoog (MS) agar media for germination. Seedlings were grown for 7 days after germination under long day conditions with 16 h light at 100 μE. Glass jars (type 742, 580 ml, WECK, Germany) were filled with modified Long Ashton medium: 0.75 mM KH_2_PO_4_, 1 mM KNO_3_, 1.5 mM Ca(NO_3_) x 4 H_2_O, 100 μM Na-Fe-EDTA, 0.5 μM CuCl_2_, 1 μM KI, 10 μM MnCl_2_ x 4 H_2_O, 0.8 μM Na_2_MoO_4_, 1.75 μM ZnCl_2_, 50 μM H_3_BO_3_, 0.1 μM CoCl_2_ x 6 H_2_O and 4.1 mM MES hydrate, supplemented with 750 μM MgSO_4_ x 7 H_2_O for control S conditions or with 50 μM MgSO_4_ x 7 H_2_O and 700 μM MgCl_2_ x 6 H_2_O for S starvation. The pH was adjusted to 7.0 with KOH. To the liquid medium 0.65% w/v Low EEO agarose (Biozym, Germany) was added prior to heat sterilization. The de-etiolated seedlings were transferred to glass jars and grown for another 16 days in a walk-in high-light growth chamber under long day conditions with 16 h light (21°C/ 50% atmospheric humidity, 500 –600 μE). The fully developed second and third oldest pairs of leaves were harvested, weighed and immediately flash-frozen in liquid nitrogen.

### Extraction of Sulfated Flavonoids

For each 1 mg fresh weight *Flaveria* sp. leaf material 40 μl of extraction solution (MeOH/H_2_O 50/50 (v/v) or 80/20 (v/v) containing 5 μM deuterated sakuranetin (as internal standard was added. To start the extraction a 300 μl aliquot of the total calculated extraction solution volume (50% MeOH) was added to the frozen plant material. The plant material was homogenized with a disposable pestle in a lobind reaction tube (Eppendorf, Germany). The remaining amount of the extraction solution was used to wash residual plant material off the disposable pestle into the reaction tube. Subsequently, the reaction tubes were sonicated for 20 min in an ice cold ultrasonic bath (Elmasonic P, Laval Lab; Quebec Canada). The cell debris was separated from the extraction solution by 15 min centrifugation with 22,000 x rpm at 4°C (Eppendorf, 5430 R). The supernatant was carefully transferred to a new lobind reaction tube. The pellets were reextracted one more time with 50% MeOH and two more times with 80% MeOH. All extracts were combined and diluted 1:30 (v/v) with MeOH for the subsequent LC-MS analyses. The stability over the time course of the extraction was confirmed by stability tests. The effect of sonication on the stability of the analytes were tested with a 500 nmol quercetin-3-sulfate solution in 80% methanol with and without 0.1% hydrochloric acid. Samples were sonicated for 30 min and the changes in the analyte concentration were analyzed. In order to test the stability of the sulfated flavonoids during the sample processing, 1 ml of 100 nmol as well as 500 nmol quercetin-3-sulfate (Q3S) solution were prepared in 60% methanol, which corresponds approximately to the final concentration in the extraction solution and left at room temperature for up to 48 h. At the beginning (0 h), and after 24 h as well as after 48 h, 100 μl of the solutions were analyzed (see Supplementary Figure S1).

### Mass Spectrometry Analysis

LC-MS profiling and quantification of the sulfated flavonoids was done with an LC-MS system based on an Agilent 1260 HPLC (Agilent Technologies, Böblingen, Germany) coupled with an AB Sciex QTrap 5500 (AB Sciex, Darmstadt Germany) linear quadrupole ion trap equipped with a Turbo V electrospray ion source. The parameters of the ion source were optimized for the analysis by direct infusion with a 500 nmol quercetin-3-sulfate solution with the same flow rate as the LC-MS system (500 μl/min).

A neutral loss scan measured in the negative mode with an offset of 80 Da was used for profiling and quantification. The 80 Da represent the mass loss of a sulfate group during the collision induced dissociation which was carried out with a collision energy of −30.0 eV. Best results were achieved using an ESI ion spray voltage of −4500 V with a heater temperature of 600°C, ion source gas 1 and 2 were set to 60 and 70, respectively, the scan rate was set to 200 Da/s.

In order to account for sample losses, which occur during the extraction, as well as for fluctuations in the device parameters an internal standard was used. deuterated sakuranetin was added to the extraction solutions in a final concentration of 5 μM prior to the extraction. As sakuranetin is itself a flavonoid, it is expected to behave similar as the sulfated flavonoids during sample preparation and analysis. The deuteration is causing a mass shift of the sakuranetin making it distinguishable from possibly natural occurring sakuranetin in the samples during MS analysis. The internal standard was detected using multi reaction monitoring (MRM). The method for the detection and quantification of the deuterated sakuranetin in positive mode is published in (Sheehan et al., 2016). The ion source parameters and the collision energy were the same as mentioned above. Two fragments were used for the MRM. One had the mass of 118 Da and the other one a mass of 168 Da.

The identification of the sulfated flavonoids was performed with an ESI QqTOF instrument (QSTAR XL, Applied Biosystems, Darmstadt, Germany). MS/MS spectra of the putative sulfated flavonoids were obtained via a nanospray offline source (Proxeon Biosystems, Denmark) and compared to the corresponding mass spectra of the respective aglycones in MassBank, if available.

Additional fragmentation experiments for structure elucidation of sulfated flavonoid-glycosides were performed with an ESI TOF instrument (Maxis 4G, Bruker Daltonics, Bremen, Germany). Sample injection was performed offline using a 1 ml gastight syringe (Hamilton, Reno, United States) with a flow rate of 7 μl/min.

### HPLC Conditions

The separation was performed on an Agilent 1260 HPLC-system (Agilent Technologies, Böblingen, Germany). Chromatographic separation was done with a Restek Raptor C_18_ Core-Shell column (3.0 × 100 mm, pore size 90 Å and a particle size of 2.7 μm). The mobile phase was H_2_O (A) and MeOH (B) with 0.1% formic acid (FA). The flow rate was 500 μl/min. The injection volume was 5 μl per sample. The gradient was: Isocratic 5% B from 0.0 to 4.0 min, linear 5–30% B from 4.0 to 6.0 min, linear 30–95% B from 6.0 to 29.0 min, isocratic 95% B from 29.0 to 35.0 min. After elution, the column was re-equilibrated with linear 95–5% B from 35.0 to 39.0 min and isocratic 5% B from 39.0 to 45.0 min. All samples were analyzed in random order.

### Quantification of Sulfated Flavonoids

The quantification of the quercetin sulfate in the samples was based on an external calibration curve from a synthetic quercetin-3-sulfate standard measured in concentrations from 10 to 750 nmol/l. As in the original samples, deuterated sakuranetin was used as internal standard for the calculation of area ratios. The concentration of the internal standard was 5 nmol/l in the calibration curve. With the calculated slope of the calibration curve the concentrations of sulfated quercetin were determined.

### Measurements of S Compounds

Sulfate was determined from ca. 50 mg lyophilized plant material by ion chromatography as described in Huang et al., 2016.

The low-molecular-weight thiols cysteine and GSH were extracted and quantified as their monobromobimane (MBB)-derivatized products from ca. 30 mg lyophilized plant material as described in Mugford et al., 2011.

Total S was determined by inductively coupled plasma mass spectrometry (ICP-MS) from 100 to 200 mg lyophilized root or shoot tissue by the Plant Metabolism and Metabolomics Laboratory, University of Cologne, using an Agilent 7700 ICP-MS (Agilent Technologies, Santa Clara, CA, United States) (Almario et al., 2017).

### Statistics and Multivariate Analysis

Statistical significance between the relative abundancies of sulfated flavonoids under control S and S starvation conditions was evaluated by the two-tailed *t*-test using Microsoft Excel 2016, with significance value of *p* < 0.05. Analytes were only included into statistical analysis if they were detected in all three biological samples under the respective conditions.

To explore the multidimensional data set of detected putative sulfated flavonoids principal component analysis (PCA) was used. The detected peak area ratios of the compounds were pareto scaled, transformed to the logarithm of base two and mean centered. Analysis was carried out using R, RStudio, and the pcaMethods package available on CRAN (Stacklies et al., 2007).

## Results

### MS Method Development

This study was conducted to develop a method to identify sulfated flavonoids qualitatively as well as quantitatively. As all sulfated flavonoids carry at least one sulfate group, it was convenient to use this specific feature (Yi et al., 2006, Lehmann et al., 2007). The sulfated flavonoids were better detectable in the negative mode, as expected from the presence of the negatively charged sulfate group. Prior to optimizing the instrument parameters, it was necessary to determine whether the loss of the sulfate group of sulfated flavonoids upon collision-induced dissociation (CID) is the main fragmentation channel. Testing 100 nmol solution of synthetic quercetin-3-sulfate in the neutral loss mode revealed that, in contrast to the anticipated neutral loss of 96 Da, a loss of 80 Da occurred corresponding to the loss of a SO_3_-group (Supplementary Figure S2). Therefore, all instrument parameters were optimized for neutral loss of the mass of 80 Da.

Parameter optimization was done via direct infusion with a 500 nmol synthetic quercetin-3-sulfate solution with a flow rate of 500 μl/min. The dynamic range and the linearity were tested by measuring a calibration curve of quercetin-3-sulfate ranging from 10 to 750 nmol. The peak intensity (counts per second) was analyzed in the range from 10 to 750 nmol. The coefficient of determination was *R*^2^ = 0.9874 (Supplementary Figure S3).

### Chromatographic Method Development

The chromatographic separation method was initially developed with quercetin-3-sulfate as reference compound and later extended to include biological samples. It turned out that standard C_18_ HPLC columns ranging from 5 to 15 cm were not able to sufficiently retain and separate the sulfated flavonoids. In contrast the use of a 10 cm Restek Raptor Core Shell column enabled separation even of the two isomers quercetin-7-sulfate and quercetin-3-sulfate (Supplementary Figure S4). The latter shows a small secondary peak, most probably from an isomer originating from the synthesis process.

### Extraction Method Development

In contrast to previous extractions based on organic solvents with addition of acids (Hannoufa et al., 1994; Phippen and Simon, 1998; Guglielmone et al., 2002; Amr and Al-Tamimi, 2007), acids were excluded here to prevent hydrolysis of the sulfated flavonoid. The extraction method was developed using leaf material of *F. robusta* and *F. australasica*. Frozen plant material was grinded, and sulfated flavonoids were repeatedly extracted with MeOH and EtOH in different concentrations (50, 80, and 100%) and signal intensities of quercetin-3-sulfate were used to compare extraction efficiency. The sample extracted with 80% MeOH showed the highest intensities of quercetin-3-sulfate in the first fractions (Table 1). In the second extraction only less than one tenth of the signal intensity was detectable. The third extraction fraction showed only signal intensities on the background noise level. However, polysulfated flavonoids are most likely better soluble in higher proportions of H_2_O. Furthermore, the evaporation rate is decreased in solutions with a high water content. The final extraction method was, therefore, chosen to be a combined extraction beginning with a 50% MeOH extraction step followed by re-extraction with 80% MeOH to achieve a complete extraction.

**TABLE 1:** Efficiency of extraction method.

**Extraction solvent**	**Methanol**	**Ethanol**
100%	3.0 × 10^5^	1.5 × 10^5^
80%	2.0 × 10^6^	1.6 × 10^6^
50%	8.5 × 10^5^	5.0 × 10^5^

*MS signal intensity of quercetin-3-sulfate (counts per s, cps) in the extraction solutions after one extraction. Methanol and ethanol were mixed with ddH_2_O.*

Since unlike flavonoids, their sulfoesters are rather unstable (Teles et al., 2018), we tested the stability of quercetin-3-sulfate in 60% methanol solution. Incubation for 48 h at room temperature did not affect intensity of the LC-MS peaks (Supplementary Figure S1), confirming stability of the sulfo-group during the extraction procedure.

### Identification of Sulfated Flavonoids in Six Species of *Flaveria*

**FIGURE 2:**
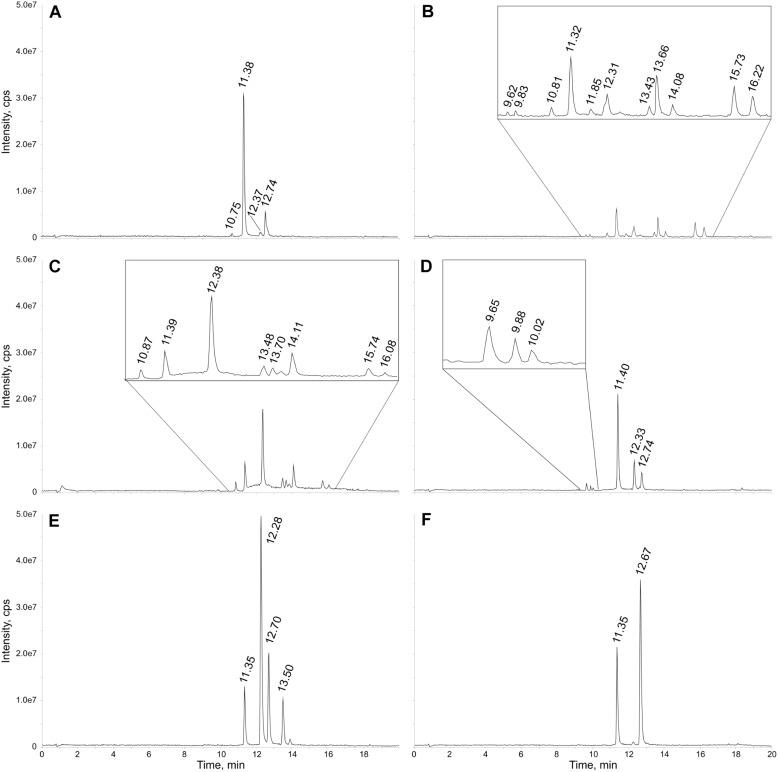
Exemplary total ion chromatograms of six *Flaveria* species grown under control S conditions. Extracts were diluted 1:30 with methanol and the injection volume was 5 μl. Peaks are labeled with their retention times. The chromatograms relate to the *Flaveria* species **(A)** *F. pringlei*, **(B)** *F. robusta*, **(C)** *F. linearis*, **(D)** *F. anomala*, **(E)** *F. australasica* **(F)** *F. bidentis*. An enlarged view is given for regions with minor peaks in the chromatograms displayed in **B–D**.

To prove the applicability of the newly developed LC-MS method for the detection of sulfated flavonoids in *Flaveria*, six species with different photosynthetic types (C_3_, C_3_-C_4_, C_4_-like and C_4_) grown under control sulfur conditions were analyzed. Representative total ion chromatograms of the LC-MS measurements show both qualitative and quantitative differences in the sulfated flavonoids in the tested *Flaveria* species (Figure 2). All peaks were assigned to flavonoids, since their masses correspond to known flavonoids plus sulfate and no signals in the mass range of other described sulfated secondary metabolites in plants (e.g., sulfated hydroxyjasmonates, phytosulfokines, or salicylate) have been detected. With the use of a synthetic standard, sulfated quercetin was identified and quantified. Further putative sulfated flavonoids were identified based on their calculated molecular weights and detected masses in the *Flaveria* extracts. *F. robusta* shows the most complex pattern of sulfated flavonoids with the highest variety of detected signals (Figure 2B). In contrast, total ion chromatograms of *F. bidentis* include only two signals (Figure 2F) and those of *F. australasica* and *F. pringlei* four (Figures 2A–E). The next step was then the identification of the detected metabolites.

For example, the representative total ion chromatogram of the *F. pringlei* extract (Figure 2A) shows four sulfated flavonoids. Peak 1 at retention time (RT) 10.75 min with m/z 557 is rather small. The mass corresponds to the mass of a sulfated isorhamnetin-glycoside. Peak 2 at RT 11.38 min has an about 30-fold higher intensity. The peak includes two different parent ions, m/z 381 and 411. The signal at m/z 381 correlates with the high-purity standard quercetin-3-sulfate. The m/z of 411 corresponds to a putative sulfated patuletin. Peak 3 at RT 12.37 min has a similarly low intensity as peak 1. This signal also includes two different components with m/z 395 and 425. The mass at 395Da corresponds to sulfated isorhamnetin and the mass of 425 Da to sulfated spinacetin/eupatolitin (14). The same m/z were detected in peak 4 at a retention time of 12.74 min, most likely corresponding to isomers of these two sulfated flavonoids, which are sulfated on different hydroxy groups of the flavonoid aglycone (see Figure 1 and Supplementary Figure S5). The intensity of this peak is about 5-fold higher than peak 3.

The detected peaks in extracts from other *Flaveria* species were similarly identified based on their m/z values and molecular weights of known sulfated flavonoids (Table 2). Alongside putative monosulfated flavonoids aglycones, putative sulfated flavonoid-glycosides were detected. In different *Flaveria* species compounds with the same m/z were detected in up to three separated peaks. Most likely those masses correspond to isomers of monosulfated flavonoids which differ in the position of the sulfation at the flavonoid hydroxy groups (see Figure 1). Altogether, the presence of at least one peak with a mass corresponding to sulfated quercetin, isorhamnetin, ombuin and patuletin was observed in all species (Table 2). Most importantly, our method detected all monosulfated flavonoids described in *Flaveria* species before (Barron et al., 1988, Hannoufa et al., 1994) and revealed their occurrence in additional species. In addition, the analysis revealed for the first time in *Flaveria* presence of sulfated flavonoid glycosides, such as isorhamnetin-sulfate glycoside (Supplementary Figure S6). The identity of the glycoside was determined through a loss of m/z 162, typical for hexoses, and comparison with known MS2 spectra of Isorhamnetin-3,7-di-O-glucoside (MassBank Record: FIO00828). Isorhamnetin-sulfate glycoside was identified previously in *Frankenia pulverulenta* (Harborne, 1975).

**TABLE 2:** List of putative sulfated flavonoids detected in six *Flaveria* species.

**Putative sulfated flavonoids**	**m/z**	**RT [min]**	***F. robusta***		**RT [min]**	***F. pringlei***		**RT [min]**	***F. linearis***		**RT [min]**	***F. anomala***		**RT [min]**	***F. australasica***		**RT [min]**	***F. bidentis***	
			***C3***			***C3***			***C3–C4***			***C3–C4***			***C4***			***C4***	
			**+S**	**−S**		**+S**	**−S**		**+S**	**−S**		**+S**	**−S**		**+S**	**−S**		**+S**	**−S**
Quercetin-SO_4_	381	11.32	x	x	11.83	x	x	11.39	x		11.38	x	x	11.35	x	x	11.35	x	x
Isorhamnetin-SO_4_	395	12.24	x		12.31	x	x	12.32		x	12.15	x	x	12.28	x	x	12.28	x	x
		12.65	x	x	12.61	x	x				12.56		x	12.70	x	x	12.67	x	x
		14.08	x	x				14.11	x										
Eupalitin-/ombuin-SO_4_	409	13.43	x	x	13.48	x	x	13.48	x	x	13.54	x		13.50	x	x	13.40	x	x
					14.15	x	x				14.90	x	x						
		15.73	x	x	15.73	x	x	15.74	x	x							15.70	x	x
								16.08	x	x		x							
Patuletin-SO_4_	411	10.80	x	x				10.87	x	x				10.88	x	x			
		11.32	x	x	11.40	x	x	11.39	x	x	11.40	x	x	11.35	x	x	11,35	x	x
Eupatolitin/spinacetin-SO_4_	425	11.85	x	x															
		12.31	x	x	12.31	x	x	12.38	x	x	12.31	x	x						
					12.73	x	x				12.74			12.70	x	x	12.67	x	x
		16.22		x															
Eupatin-SO_4_	439	13.66	x	x	13.71	x	x	13.70	x	x	13.74	x					13.74	x	x
		13.95	x	x	13.89	x		13.88	x	x	13.89	x		13.90	x	x			
Quercetin-SO_4_-glycoside	543				9.53	x	x												
					10.42	x	x										10.55	x	x
														12.28	x	x			
Isorhamnetin-SO_4_-glycoside	557	9.88	x								9.88	x	x	9.88	x	x			
		10.55		x	10.75	x	x										10.72	x	
								11.88	x	x									
								12.61	x	x									
Patuletin-SO_4_-glycoside	573	9.63	x	x	9.62	x	x	9.80	x	x	9.65	x	x						
					10.55	x											10.54	x	
Eupatoletin-/spinacetin-SO_4_-glycoside	587										10.02	x	x	9.98	x				
								10.70	x	x									
								11.23	x	x									

*Putative sulfated flavonoids have been identified through detected molecular masses and compared to sulfated flavonoids described previously in *Flaveria* (Barron et al., 1988). Shown are compounds detected in leaf extracts of plants grown under control sulfur conditions (+S) and low sulfur conditions (**−**S) with the retention times (compare Figure 2).*

### Relative Quantification of Sulfated Flavonoids

**FIGURE 3:**
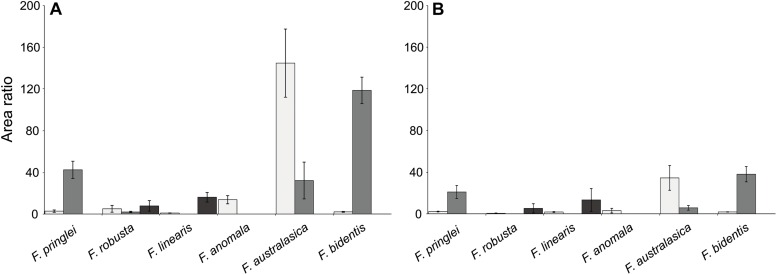
Area ratios of putative isomers of sulfated isorhamnetin in six *Flaveria* species grown in control and low S conditions. **(A)** Area ratios of the plant samples grown in control S condition. **(B)** Area ratios of the plant samples grown under low S condition. Different colors represent different sulfated isorhamnetin isomers. Data are shown as mean values and standard deviation from 3 biological replicates.

To enable relative quantification, of the putative sulfated flavonoids with the same retention time, an internal standard (deuterated sakuranetin) was used in all extracts. Such quantification was used to assess the effect of S starvation on the sulfated flavonoids. The analysis showed that, the qualitative patterns of sulfated flavonoids did not change under S starvation (Figure 2 and Supplementary Figure S7). The same was true, e.g., for the three isomers of sulfated isorhamnetin (Figure 3). Significantly, for most of these isomers the relative abundances were lower in the S starved samples than in the control samples (Figure 3). The reduction in sulfated isorhamnetin was the highest in *F. australasica* and *F. bidentis* and rather low in *F. pringlei* and *F. linearis* (Figure 3).

**FIGURE 4:**
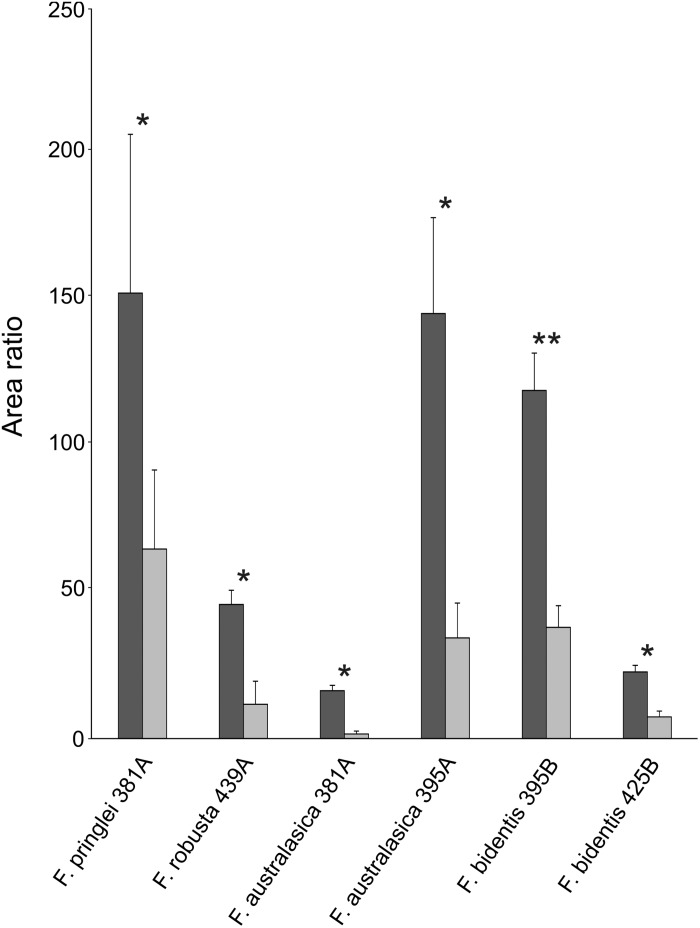
Sulfated flavonoids affected by S starvation. Relative abundances of sulfated flavonoids with significant differences between control S (dark gray) and low S (light gray) grown *Flaveria* species. Data are shown as mean values and standard deviation from 3 biological replicates. Asterisks represent significance levels calculated by a two-sided student’s *t*-Test (^*^p < 0.05; ^∗∗^*p* < 0.005). Characters A and B in the *x*-axis labels indicate putative isomers with different retention times.

Similar to isorhamnetin, several other putative sulfated flavonoids show significant differences in the average area ratios of control sulfur and S starvation samples (Figure 4). In all cases area ratios of the control sulfur samples were higher than those of their counterparts in S starved ones. In *F. australasica*, significant differences were observed for isomers of sulfated quercetin (381 Da) and sulfated isorhamnetin (395 Da/earliest retention time). Sulfated quercetin was reduced also in S starved samples of *F. pringlei* and sulfated isorhamnetin in *F. robusta*. Furthermore, a significant difference was observed for the putative sulfated eupatin (439 Da) in *F. robusta* and putative sulfated spinacetin/eupatolitin (425 Da) in *F. bidentis*. Thus, many sulfated flavonoids are less abundant in S starved plants than in those with control nutrition, in agreement with the general regulation of S containing secondary metabolites.

### Absolute Quantification of Sulfated Quercetin

**FIGURE 5:**
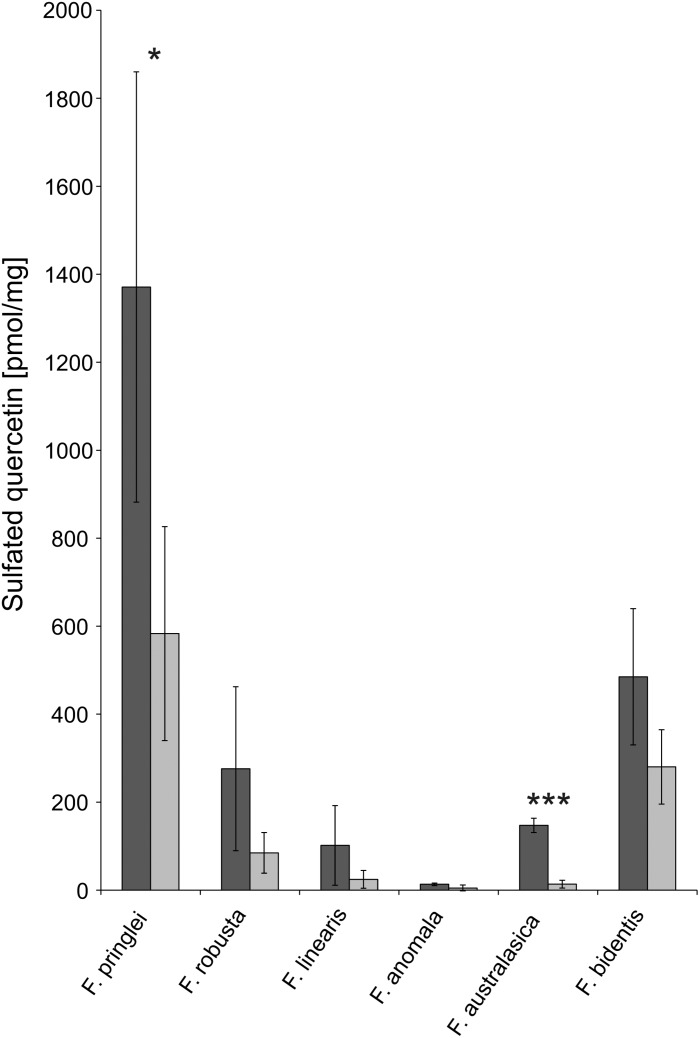
Absolute concentrations of sulfated quercetin in *Flaveria* species. Quercetin-3-sulfate was determined using the new LC-MS procedure in 6 *Flaveria* species grown at control (dark gray) or low S (light gray) supply. Data are shown as mean values and standard deviation from 3 biological replicates. Asterisks represent significance levels calculated by a two-sided student’s *t*-Test (^*^p < 0.05; ^∗∗^*p* < 0.005; ^∗∗∗^*p* < 0.0005).

One aim of the new developed LC-MS method for sulfated flavonoids was to enable absolute quantification, which would allow to assess their significance as S-pool in *Flaveria*. This has been achieved by having an internal standard during extraction and by calibration of the LC-MS with a synthetic quercetin-3-sulfate standard. In all species, higher concentrations of quercetin-3-sulfate were detected in samples of plants grown on control sulfate medium compared to those grown on low sulfur medium, but due to high variation, these differences were not always significant. The highest concentration of quercetin-3-sulfate was observed in *F. pringlei* under control S conditions (Figure 5). In *F. anomala* the concentration was the lowest of the tested *Flaveria* species, more than 100-fold lower than in *F. pringlei.* The same was true for the S starved plants, the highest concentration of quercetin-3-sulfate was found in *F. pringlei* and the lowest in *F. anomala* (Figure 5).

**FIGURE 6:**
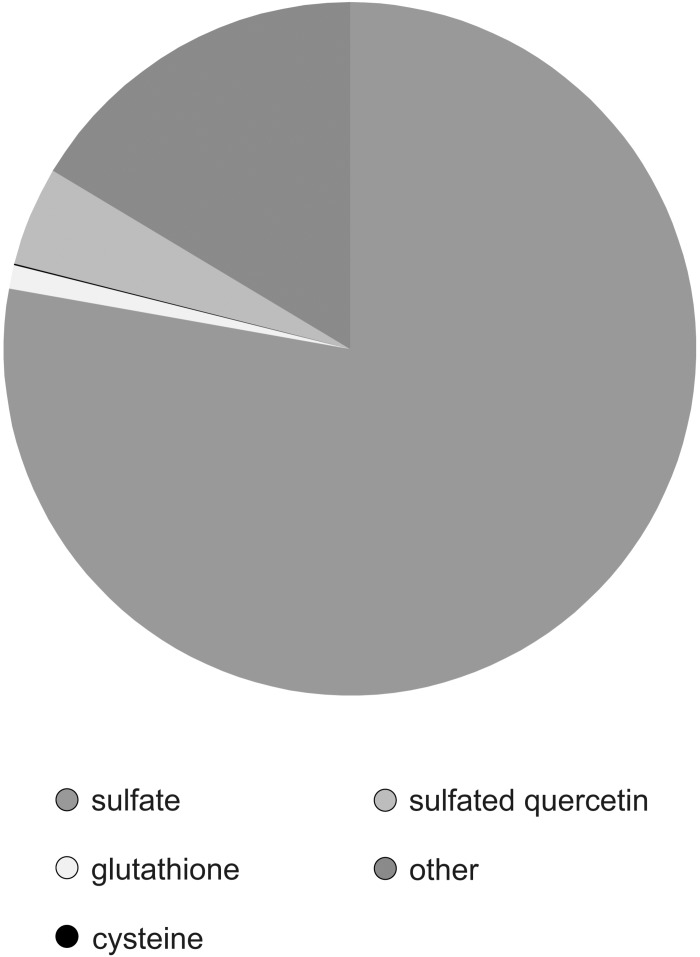
Partitioning of S in different pools in leaves of *F. pringlei*. Total S, sulfate, glutathione, cysteine, and sulfated quercetin were measured in leaves of *F. pringlei*. Data are shown as percentage of S in the individual compounds.

To determine the partitioning of sulfur to sulfated flavonoids and other pools, we determined various sulfur compounds in the leaves of *F. pringlei*. Total sulfur amounted to 29.7 ± 2.9 μmol/g FW. Sulfate was the most abundant sulfur compound amounting to 23.1 ± 2,1 μmol/g FW, i.e., 77.8% (Figure 6). The thiols glutathione and cysteine formed with 0.33 μmol/g FW and 0.016 μmol/g FW 1.1 and 0.05%, respectively, of total S. The concentration of quercetin-3-sulfate in *F. pringlei* was 1.37 μmol/g FW, and so with 4.6% ca. four-fold higher than the concentration of glutathione (Figure 6). Therefore, sulfated flavonoids represent a significant S pool in *Flaveria* and have to be taken into account in studies of regulation of S homeostasis in these plant species.

## Discussion

The aim of the study was to develop a specific and quantitative method to analyse sulfated flavonoids, in order to address the function of these metabolites in plants. We have optimized the extraction protocol and developed an LC-MS method based on a neutral loss scan and thus could show differences in the sulfated flavonoid profiles of six tested *Flaveria* species.

### Method Evaluation

Since previous methods aimed toward qualitative analysis of sulfated flavonoids in different species, it was necessary to develop a method which allowed quantitative analysis together with a qualitative one. The methanol and sonication based extraction protocol enabled to extract sulfated flavonoids from approximately 30–100 mg *Flaveria* leaf material in a quantitative matter. With the introduction of an internal standard and a weight dependent extraction procedure it was possible to quantify sulfated flavonoids in leaf material relatively. An absolute quantification is possible in the same way when the respective standard is available. Previous methods suffered from the need of higher amount of initial plant material. Additionally, they demanded more complex sample preparation procedures than grinding and sonication. For example, in the study of Hannoufa et al., 1994, samples were first defatted with hexane, followed by an extraction with ethyl acetate. Extracts were then subjected to TLC, the individual metabolites were scraped off the plates and characterized by UV spectra in methanol, after addition of shift reagents, and via chromatography. Therefore, we aimed to circumvent these complications using modern MS methods for analyzing the plant extracts. In proteomics it is a common technique to use neutral loss scan-based methods to detect phosphorylation of specific peptides (Lehmann et al., 2007). We adapted this method for detection of sulfated flavonoids.

Indeed, a neutral loss scan MS method, based on collision induced dissociation of the flavonoids sulfate group, turned out to be an efficient and reproducible approach for the identification and quantification of these compounds. The method decreases the complexity of the detected spectra because almost no compounds without sulfate are detected. This reduction thus allows identification and quantification of sulfated flavonoids and other sulfated compounds on instruments with lower sensitivity and specificity due to the less complex spectra. Electrospray ionization is a rather soft ionization method; nonetheless source parameters have to be chosen carefully as small changes in the instrument parameters drastically affected the signal intensity and quality, which could lead to a loss of information due to in source decay of compounds of interest.

With a parallel recorded MRM experiment it was possible to introduce deuterated sakuranetin as internal standard. This allows the normalization of samples, which enables comparability and relative quantification of different samples.

As reported in previous studies, up to 7 isomers of the same flavonoid aglycone can be present in one species (Barron et al., 1988; Harborne and Williams, 2000). Therefore, a direct infusion MS approach was excluded as this would not allow the detection of isomers with identical masses. With the C_18_ Core Shell column a sufficient separation of the isomers was possible and verified using synthesized standards quercetin-3-sulfate and quercetin-7-sulfate. Indeed, using HPLC coupled to an MS, isomers sulfated on different positions of the flavonoid and a sulfated flavonoid-glycosides were detected (Supplementary Figures S4–S6).

The analysis of six different *Flaveria* species revealed highly different patterns of sulfated flavonoids. These compounds have been detected in several *Flaveria* species before, using TLC, HPLC-UV/VIS and chromatography methods (Varin et al., 1986; Barron et al., 1988, Hannoufa et al., 1994). With the neutral loss scan based method, not only we identified these patterns in a single experiment but we also achieved a relative quantification of the detected sulfated flavonoids together with an absolute quantification of quercetin-3-sulfate within this single experiment.

Our LC-MS method verified the previously reported occurrence of single sulfated quercetines and isorhamnetins in *F. bidentis* (Hannoufa et al., 1994). In addition to that, however, we could identify masses corresponding to mono sulfated ombuin, patuletin, eupalitin, spinacetin/eupatulitin, eupatin, extending thus the sulfoflavonoid profile of this species. In *F. linearis* monosulfated quercetins, isorhamnetins and patuletin were identified in the past (Hannoufa et al., 1994). Again, these sulfated flavonoids were detected and in addition also masses corresponding to monosulfated ombuin, eupalitin, spinacetin/eupatulitin and eupatin. In *F. pringlei* the presence of monosulfated quercetins and patuletin were observed in past studies (Hannoufa et al., 1994) and confirmed by our analysis. We additionally report on the detection of masses corresponding to monosulfated isorhamnetins, ombuin, eupalitin, spinacetin/eupatolitin, and eupatin. Novel compounds identified by this approach are sulfoflavonoid glycoside, which were described for the first time in *Flaveria*.

Thus, the new LC-MS method enables detection of a higher variety of different sulfated flavonoids in *Flaveria* than it has been achievable with previous methods. In the same analysis it is also possible to quantify these flavonoids if a high purity analytical standard is available. However, in contrast to previously published studies on sulfated flavonoids in the genus *Flaveria* we were not able to observe the presence of polysulfated flavonoids. Possibly the level of polysulfated flavonoids in the plants is under detection limit in the complex sample due to matrix effects. It is also possible that the neutral loss of the polysulfated flavonoids during CID is not 80 Da, since more than one sulfate group might be lost during the process. A neutral loss scan with an offset of a mass of 160 and 240 Da was tested but no previously undetected compounds could be observed in these approaches.

### Sulfated Flavonoids in *Flaveria*

**FIGURE 7:**
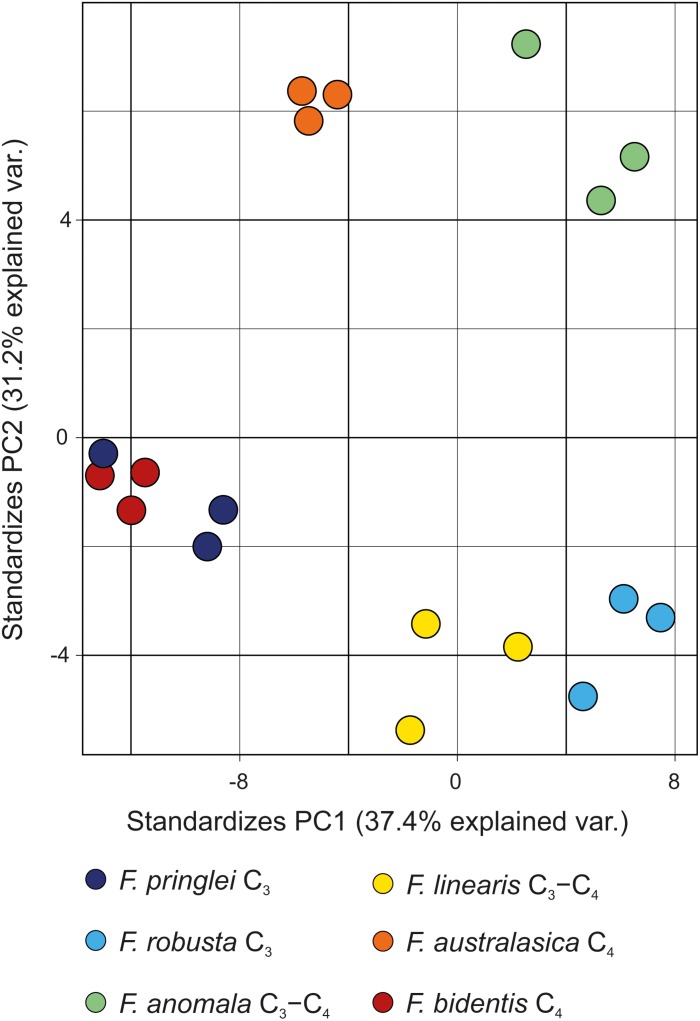
Score plot of the PCA model for the six *Flaveria* species grown under control S conditions. For the PCA the area ratios of all detected compounds were used. Each circle represents one individual sample.

We set to develop the analytic method in order to answer several important questions on sulfated flavonoids in *Flaveria*. Firstly, we compared the qualitative sulfated flavonoids profiles in six species to test whether there is any correlation with photosynthesis type. In all analyzed species the number of detectable isomers of monosulfated flavonoid aglycones was highly variable. However, as reported previously with smaller samples (Barron et al., 1988; Hannoufa et al., 1994), there is no visible relation between the profiles of monosulfated flavonoids and the photosynthetic type (Figure 7). Interestingly, a higher variety of multiple isomers was detectable in the C_3_-species (*F. robusta* and *F. pringlei*).

Second question related to regulation of accumulation of sulfated flavonoids by S starvation. As expected, at low sulfur conditions the number of detected compounds was in most cases decreasing as some of the signals were below the detection limit. This was further corroborated by the results of the relative quantification in the six species. A clear trend toward a lower concentration in S starved leaves was visible for all sulfated flavonoids, even if significance levels were not always reached. An independent absolute quantification of the quercetin sulfate confirmed this conclusion (Figures 4, 5). Thus, sulfur availability has an influence on the sulfated flavonoid concentrations in the plants. This is similar to the other class of sulfated metabolites, the glucosinolates in *Arabidopsis* (Hirai et al., 2005). Accumulation of glucosinolates upon S starvation is controlled primarily by blocking the transcription of the biosynthetic genes through SDI proteins (Aarabi et al., 2016). The mechanisms for reduction of sulfated flavonoids in *Flaveria* are not known but it would be highly interesting to compare with those controlling glucosinolates in *Arabidopsis*.

The third question on sulfated flavonoids in *Flaveria* is a quantitative one, which could not be addressed without a quantitative analytical method. For further studies of S metabolism, it is imperative to know how important sulfated flavonoids are for S balance in *Flaveria*, particularly in comparison with *Arabidopsis*, where glucosinolates form a significant S pool comparable in size with glutathione (Mugford et al., 2011). In *F. pringlei* the concentration of sulfated quercetin reached 1.37 μmol/g FW. This concentration is ca. four-fold higher than glutathione and 85-fold higher than cysteine. The sulfated flavonoid profile in *F. pringlei* shows one large peak containing both sulfated quercetin and patuletin and three much smaller peaks containing four other compounds. Thus, it is impossible to say, what portion of the total sulfated flavonoid pool is formed by the quantified sulfated quercetin. But even if the other compounds would not add substantially to the total concentration of sulfated flavonoids, they are a very significant pool of sulfur that needs to be taken into account in studies on sulfur nutrition in *Flaveria*.

The high concentration of sulfated quercetin in *F. pringlei* indicates that it may serve as a storage of sulfate. The sulfate might be remobilized by enzymatic reaction catalyzed by sulfatase, which is present in bacteria as well as humans and animals (Dodgson and Rose, 1979). However, to date sulfatase has not been detected in plants. Whether other enzymes might release sulfate from the sulfated compounds, e.g., the biosynthetic sulfotransferases remains to be established. Another possible function of sulfated flavonoids could be an overflow mechanism for the stabilization of the plants sulfate homeostasis during excessive sulfate supply.

## Conclusion

We developed and tested a new quantitative LC-MS method for extraction and analysis of sulfated flavonoids from plants. The method enabled us to describe the metabolic variation of this class of metabolites in *Flaveria*, which is however, not correlated with photosynthetic type. We could show that sulfated flavonoids are an important pool of sulfur, and that their concentration in leaves declines in sulfur deficient plants. The new method reported here will enable to quantify sulfated flavonoids in other plants and provide the means to address various research questions on this enigmatic class of plant metabolites. It can easily be adapted to analyze a more general group of sulfated secondary metabolites.

## Author Contributions

NK and FB conducted most of the experiments, analyzed the results, prepared the figures, and wrote most of the manuscript. SG grew the plants and conducted measurements of S compounds. SK and SM conceived the idea for the project. All authors were involved in discussions and writing the manuscript.

## Conflict of Interest Statement

The authors declare that the research was conducted in the absence of any commercial or financial relationships that could be construed as a potential conflict of interest.
