# Supplementary material for: A Novel Method for Identification and Quantification of Sulfated Flavonoids in Plants by Neutral Loss Scan Mass Spectrometry
Source: Front Plant Sci. 2019 Jul 5;10:885. doi: 10.3389/fpls.2019.00885 (PMC6625178; doi:10.3389/fpls.2019.00885)
Supplement: Supplementary file 1 [file Data_Sheet_1.PDF]

SUPPLEMENTAL INFORMATION FOR THE MANUSCRIPT

A novel method for identification and quantification of sulfated flavonoids in plants by neutral loss scan mass spectrometry

Niklas Kleinenkuhnen, Felix Büchel, Silke C. Gerlich, Stanislav Kopriva, Sabine Metzger

SUPPLEMENTAL FIGURES

Figures S1-S7

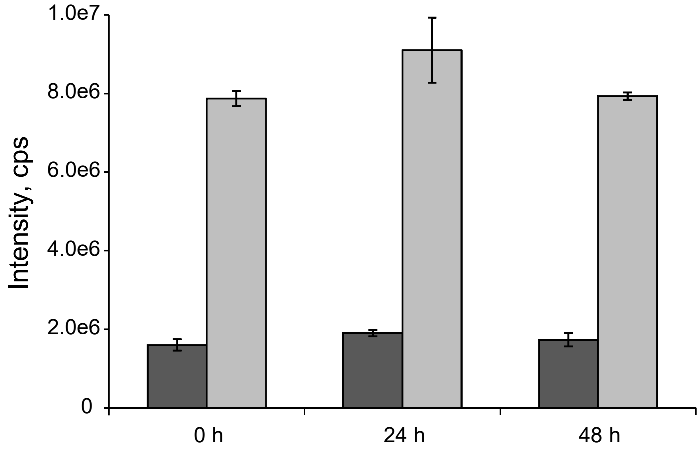

Figure S1: Stability test of quercetin-3-sulfate over a time course.
The stability over the time course of the extraction was confirmed by stability tests with quercetin-3-sulfate (Q3S). In order to exclude possible sample degradation during sample processing, 1 ml of 100 nM and 500 nM Q3S solution were prepared in 60% methanol and left at room temperature for up to 48 hours. At the beginning (0 h), and after 24 and 48 hours, 100 µl of the solutions were analysed with LC-MS.

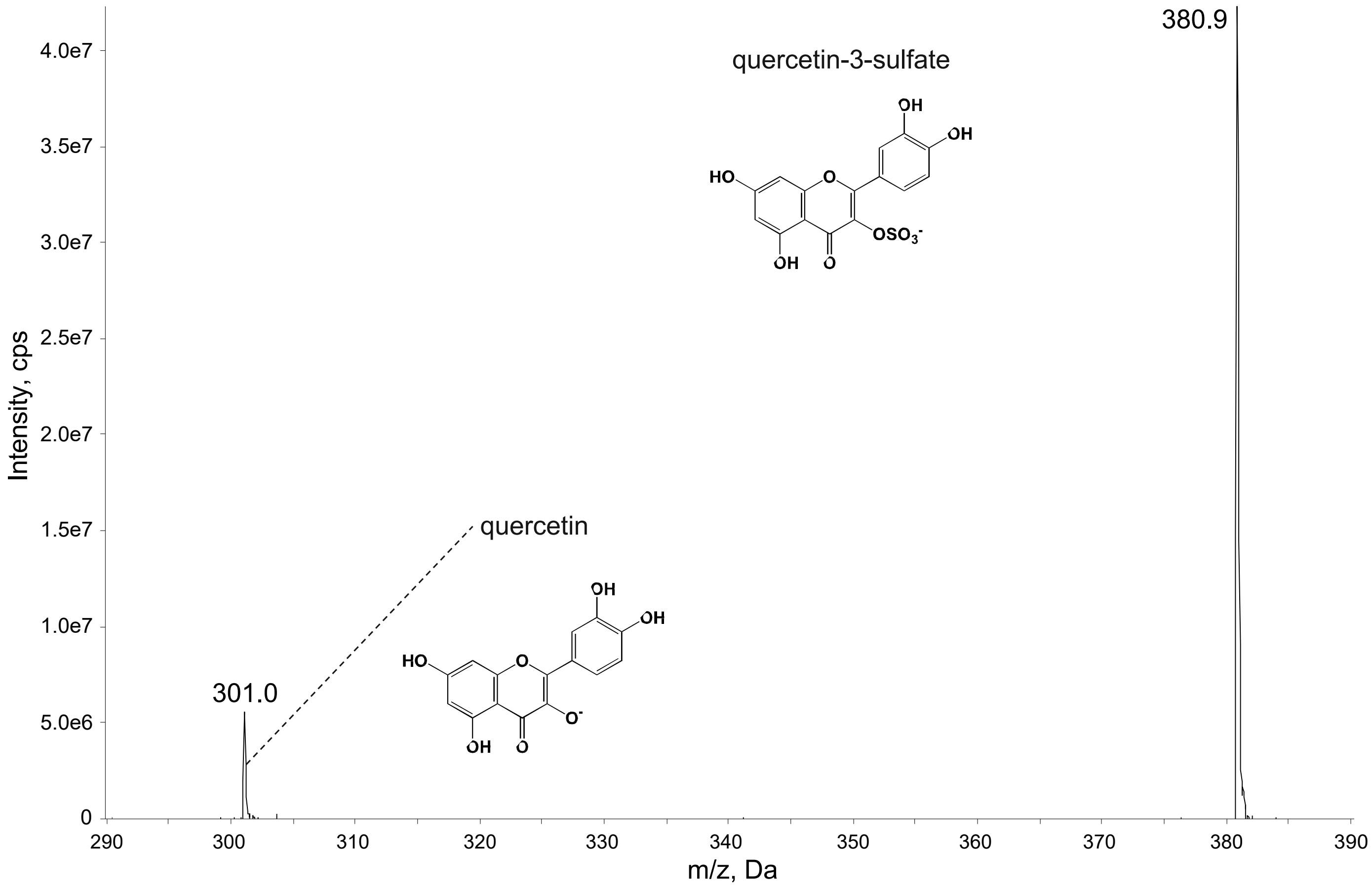

Figure S2: Representative MS2 -spectra of synthetic quercetin-3-sulfate.
A 500 nmol solution of synthetic quercetin-3-sulfate was analyzed in a direct infusion MS2-experiment. The mass loss of 80 Da corresponds to neutral loss of the SO_3^--group. The ion with the m/z of 301 corresponds to the flavonoid aglycone quercetin.

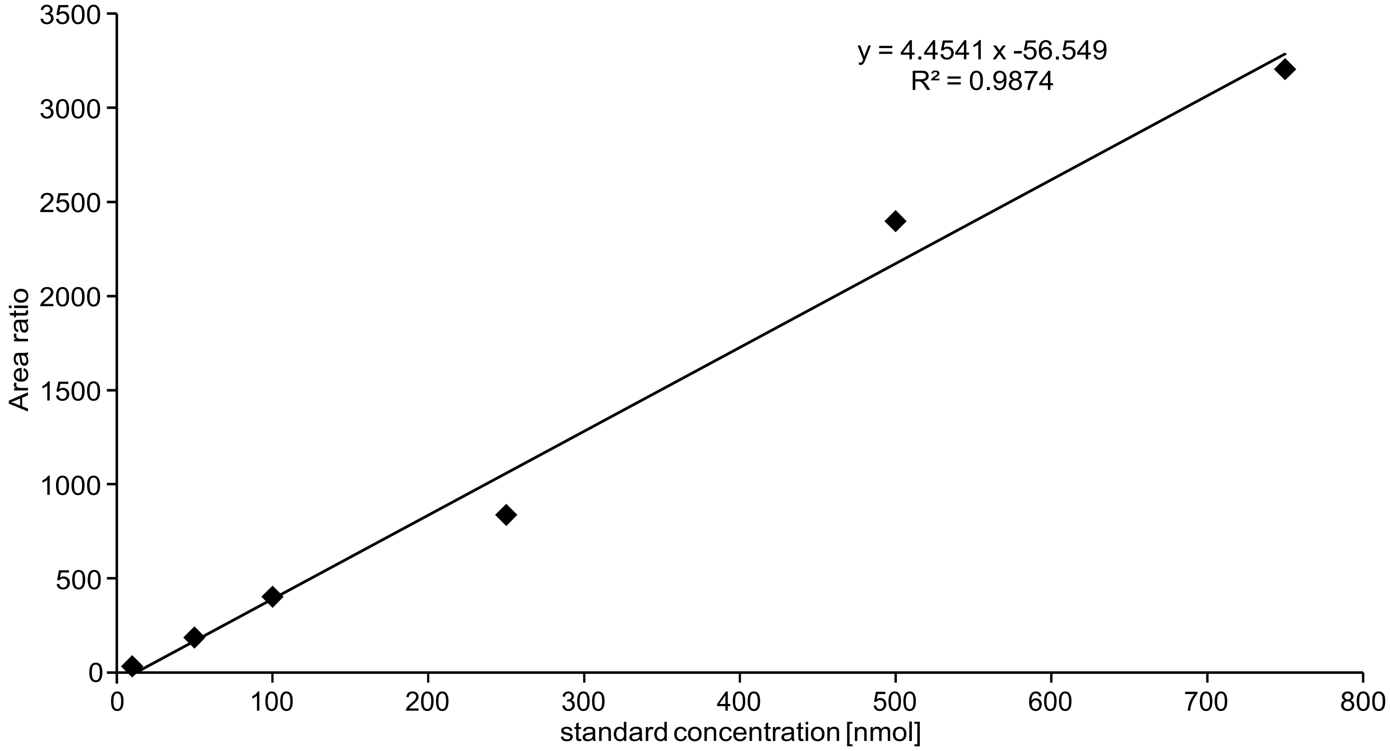

Figure S3: Calibration curve of six Q3S standard solutions.
The calibration curve was measured in the Neutral Loss Scan mode using the Restek Raptor C18 Core-Shell column. The used concentrations were 10 nmol, 50 nmol, 100 nmol, 250 nmol, 500 nmol and 750 nmol with deuterated Sakuranetin as an internal standard. Coefficient of determination and just equation are given. Area ratio is defined as the ratio of the peak area of the target compound to the peak area of the internal standard.

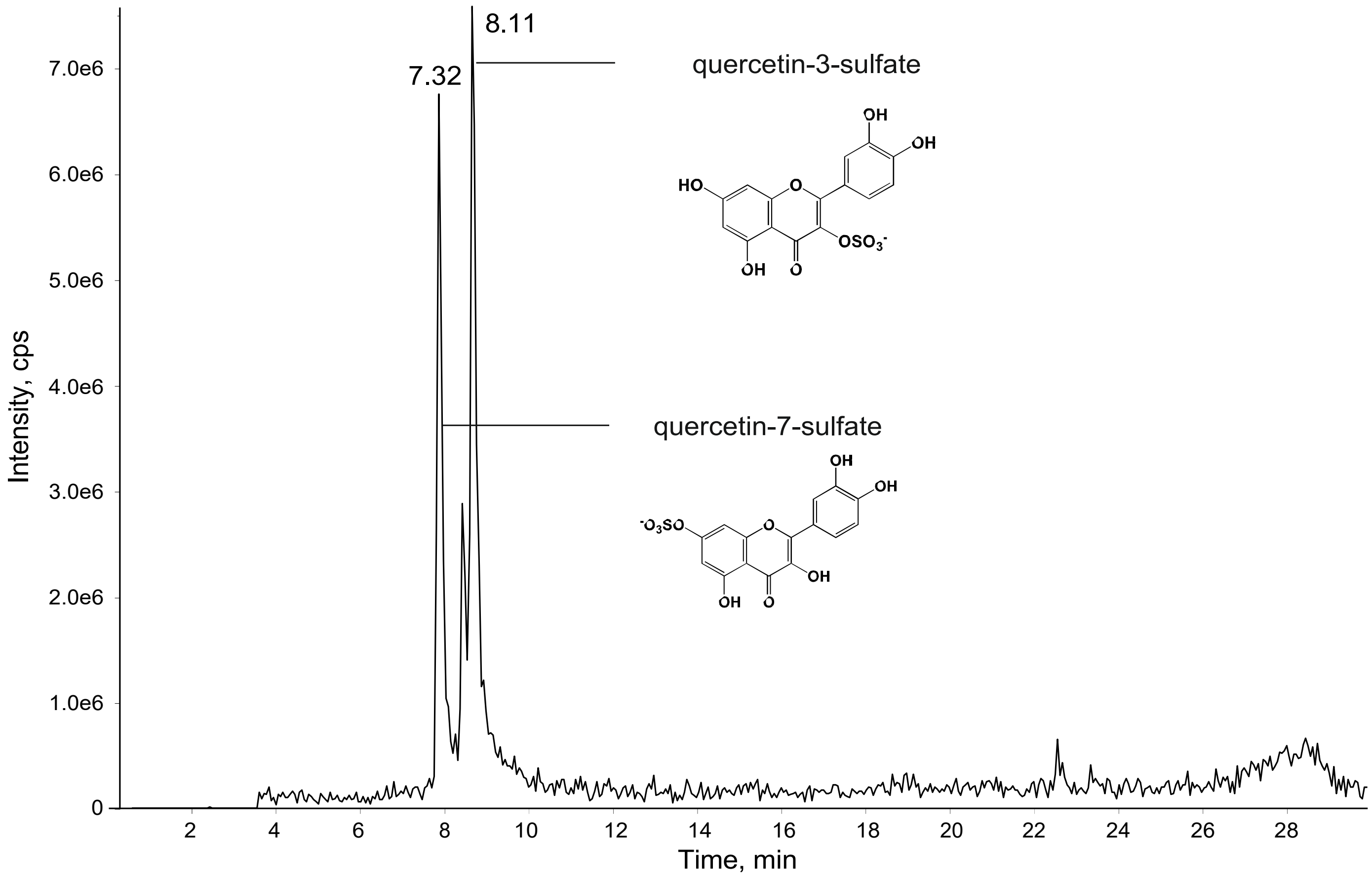

Figure S4: Total ion chromatogram of the neutral loss scan of a 250 nmol Q3S and Q7S solution. Chromatographic separation was done with a Restek Raptor C18 Core-Shell column (3.0 x 100 mm, pore size 90 Å and a particle size of 2.7 µm). The mobile phase was H_2O (A) and MeOH (B) with 0.1% formic acid (FA). The flow rate was 500 µl/min. The injection volume was 5 µl per sample. The gradient was: Isocratic 5% B from 0.0 – 4.0 min, linear 5-30% B from 4.0 - 6.0 min, linear 30 – 95% B from 6.0 – 29.0 min, isocratic 95% B from 29.0 – 35.0 min.

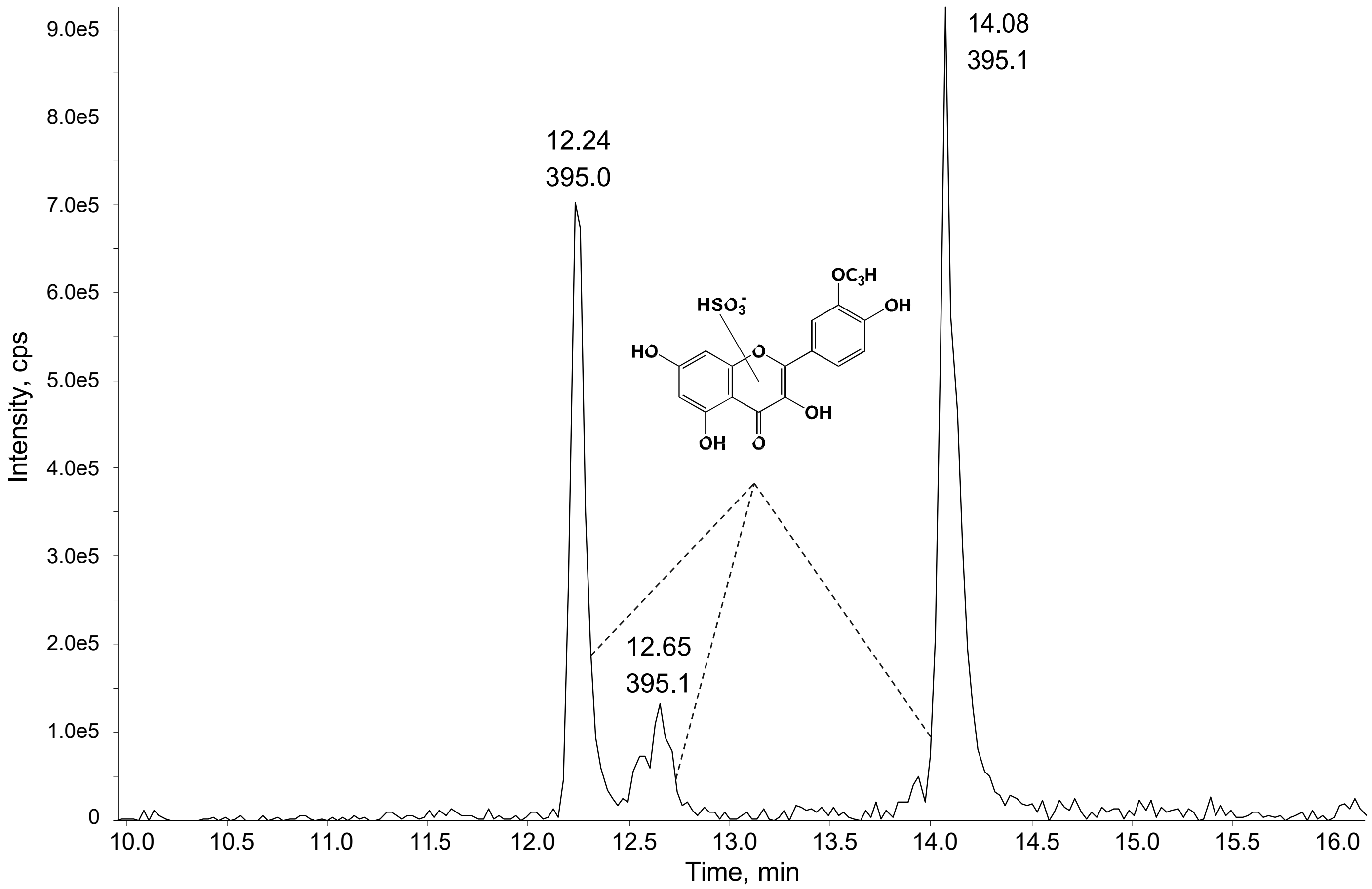

Figure S5: Extracted ion chromatogram of the neutral loss scan of three sulfated isorhamnetin isomers in an extract of *F. robusta*.

The same m/z = 395 were detected in all three peaks corresponding to isomers of the sulfated isorhamnetin, which are sulfated on different hydroxy groups of the flavonoid aglycone.

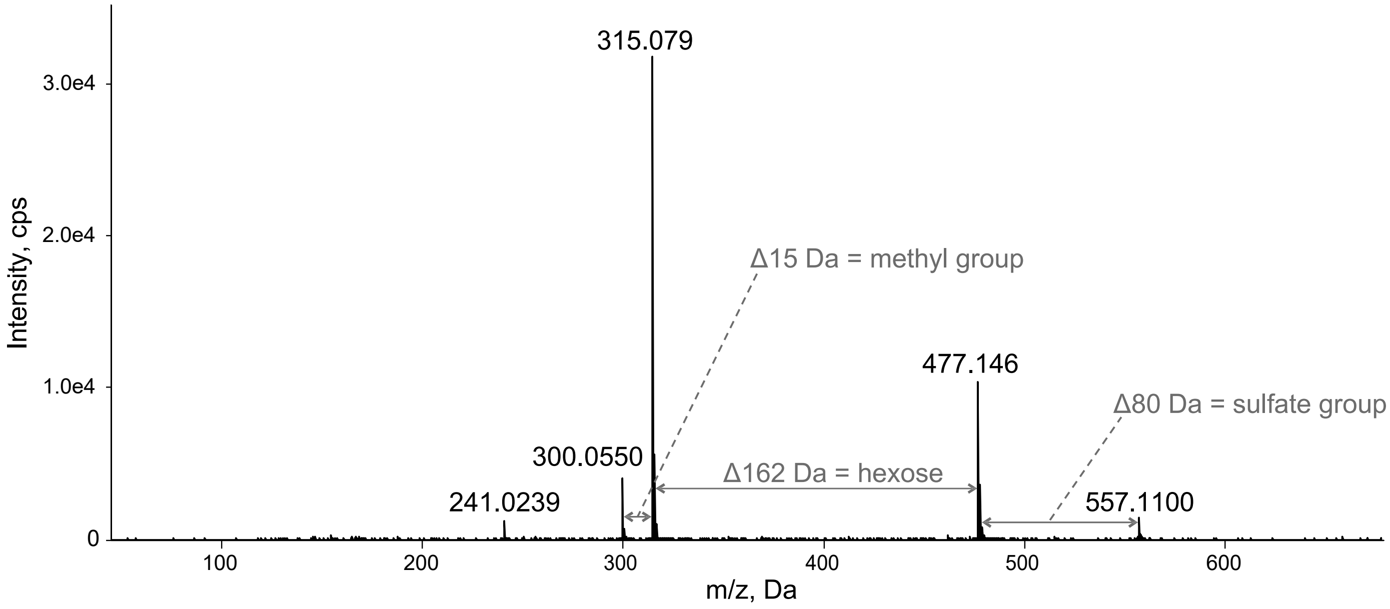

Figure S6: ESI-MS/MS spectrum of a putative sulfated isorhamnetin-glycoside (m/z = 557) in negative ionization mode.

The spectrum was generated with an LC-MS System (ESI-Q-TOF, Maxis 4G, Bruker, Germany and a Dionex Ultimate 3000 HPLC, Thermofisher, USA). The fragment spectrum of m/z = 557 show clearly a mass loss of 80 Da corresponding a sulfate group and an additional loss of 162 Da corresponding a hexose. The highest fragment signal m/z 315 corresponds to the mass of isorhamnetin. Additionally to the loss of a sulfate group and a hexose, isorhamnetin specific fragments were detected, which can be compared to the fragment spectra from MassBank.eu. This is an indication that the ion with an m/z = 557 is a glycosylated isorhamnetin-sulfate.

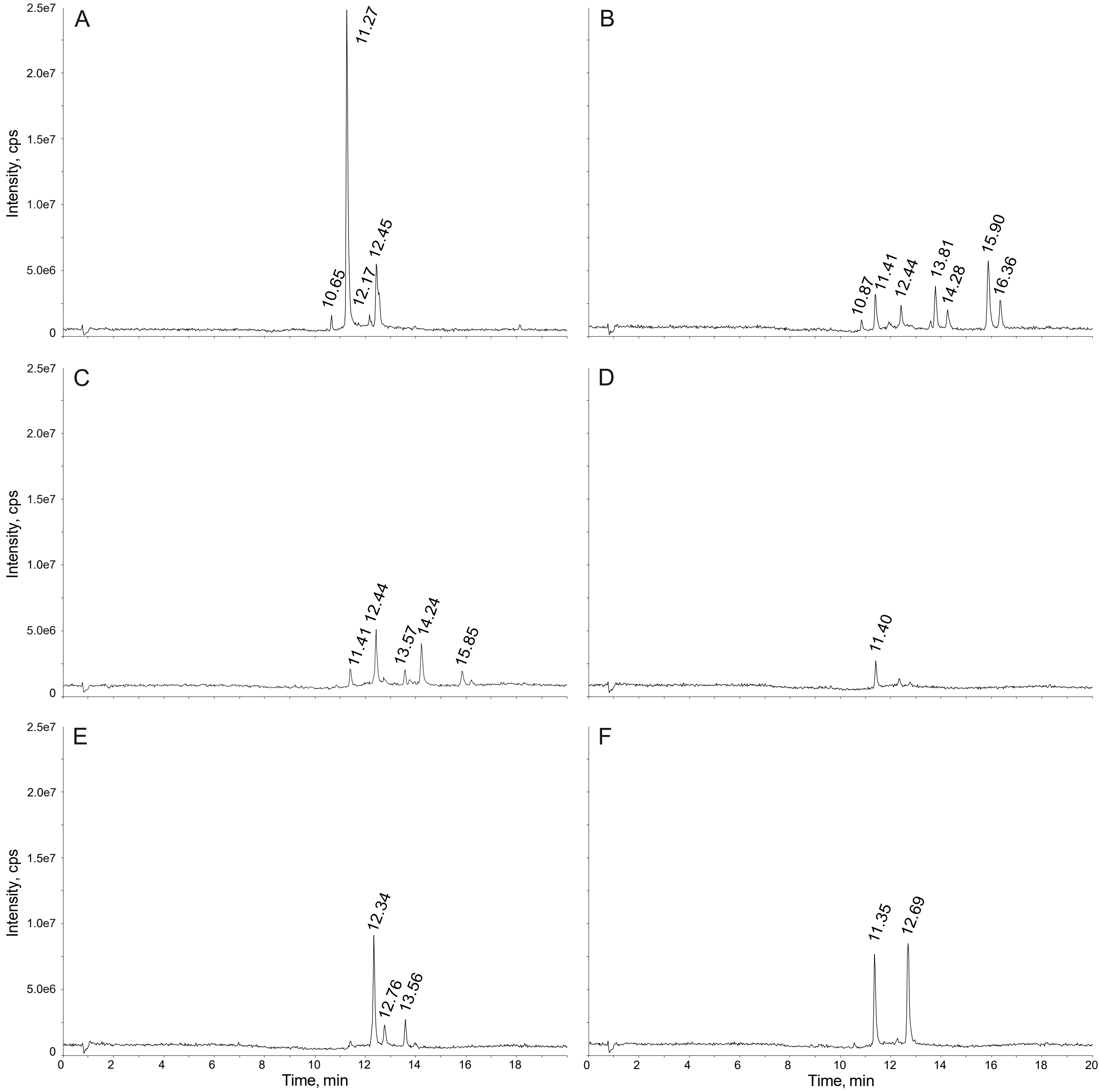

Figure S7: Exemplary total ion chromatograms of six *Flaveria* species grown under low sulfur conditions.

Samples were diluted 1:30 with MeOH and the injection volume was 5 µl. Peaks are labelled with their retention times. The chromatograms relate to the *Flaveria* species (A) *F. pringlei*, (B) *F. robusta*, (C) *F. linearis*, (D) *F. anomala*, (E) *F. australasica*, (F) *F. bidentis*.
